# Conceptual Design and Numerical Validation of a Carbon-Based Ink Injector

**DOI:** 10.3390/ma16196545

**Published:** 2023-10-03

**Authors:** Arleth Ortega-Gutiérrez, Job Eli Escobar-Flores, Mario Alberto Grave-Capistrán, Noé López-Perrusquia, Marco Antonio Doñu-Ruiz, Armando Oropeza-Osornio, Christopher René Torres-SanMiguel

**Affiliations:** 1 Grupo Ciencia e Ingeniería de Materiales, Universidad Politécnica del Valle de Mexico, Estado de Mexico 54910, Mexico; 2 Instituto Politécnico Nacional, Centro de Estudios Científicos y Tecnológicos N° 2 “Miguel Bernard”, Ciudad de Mexico 11200, Mexicomgravec1200@alumno.ipn.m (M.A.G.-C.); 3 Unidad Zacatenco, Sección de Estudios de Posgrado e Investigación, Escuela Superior de Ingeniería Mecánica y Eléctrica, Instituto Politécnico Nacional, Ciudad de Mexico 07738, Mexico; mgravec1200@alumno.ipn.m; 4 Unidad Ticomán, Escuela Superior de Ingeniería Mecánica y Eléctrica, Instituto Politécnico Nacional, Ciudad de Mexico 07340, Mexico

**Keywords:** 3D printing, carbon nanotubes, CFD, nano ink

## Abstract

This paper shows the design of an injector, using carbon nanotubes as inkjet material, implemented in a 3D printer. According to the available literature, few injectors are capable of depositing material. Due to the lack of information, the central part of this research is to develop a suitable device for ink injection that is capable of applying the Fused Deposition Modeling (FDM) method to print nanomaterial ink. The injector was designed using a CAD program based on an open-source desktop 3D printer, which allows it to be modified according to the needs of the injector. This prototype was manufactured in aluminum alloy 7075T6. Computational fluid dynamics (CFD) were carried out to analyze the behavior of the fluid when it passes through the injector, obtaining parameters such as pressure, velocity, and vorticity. An experimental matrix of the injector operation was carried out to achieve an adequate printing speed. The results show that the optimum speed was 250 ms, considering that a temperature of 100 °C is needed in the heated bed to dry the ink so that it does not undergo expansion.

## 1. Introduction

In 1990, the Massachusetts Institute of Technology developed the first 3D printer using inkjet [1]. Three-dimensional printing, also called additive manufacturing, is a process that allows the creation of three-dimensional objects. The 3D model is designed using a CAD program and is developed by depositing materials through the superposition of layers of material until the final product is obtained [2,3,4,5]. The 3D printing process runs openly. That is, the number of layers to be built and the places where the droplets will be deposited in each layer will be determined in advance and will not change during the printing process [6,7,8].

The most widely used technology for inkjet is direct 3D printing, also known as binder injection. The injection process consists of solidifying the layer, removing excess dust, and lowering the platform to allow a new bed of dust to be deposited and leveled [9,10]. Bioplotter 3D printing, characterized by using bio-ink as printing material, uses a pneumatic pressurized air system to place the bio-ink layer by layer. [11,12]. In the inkjet process, droplets are generated with a diameter between 50 and 500 µm and are variable depending on the nozzle size, the type of printing and printing material, among other factors [13,14]. The small size of the drop influences the impression since a high resolution depends on this [15]. The inkjet process is ejected directly onto the paper surface from the inkjet nozzles, and the ink water is absorbed on the paper surface [16,17]. Photopolymer inks consist of a linear motor that transports the substrate, a nozzle that releases drops, and an ultraviolet (uv) drying system for the deposited drops [18,19]. Different ink-printing methods are used to create sensors or electronic components; most existing devices use the inkjet print head. Patent US20120170171A1 [20] describes a technique for printing flexible electronic components printed by inkjet. It consists of depositing graphene oxide flakes on a substrate in ink using an inkjet printer. This is used to make graphene electrodes. The document KR20130075512A uses a method to fabricate a flexible graphene thin film electrode [21]. The document KR101251216B1 explains the application of graphene production with transparencies through an electrode using inkjet printing based on a solution of nanoparticles [22]. Document US20160067891A1 shows a method for forming air gel by mixing graphene oxide powder with a solution to create an ink. A 3D printing technique can deposit the ink [23].

Different types of ink printing methods are made differently, using components according to the needs [24]. However, ink is commonly ejected using a printer using the print head. This research aims to develop a low-cost injector design that employs carbon nanotube ink as print material with features such as conductivity and flexibility to provide different engineering applications. This device will be helpful in developing sensors, and it is easy to adapt to a commercial 3D printer. Carbon nanotubes present physical properties such as density, mechanical resistance, thermal conductivity, flexibility, and electrical features. Due to its dimension of 50 nm (carbon nanotubes), it is considered nanomaterial; furthermore, this injector is designed to print any nanomaterial such as graphene. 

## 2. Materials and Methods

### 2.1. Carbon-Based Ink Preparation

For the production of the carbon-based ink, 9.3 mg of carbon nanotubes, 800 µL of ethanol, 150 µL of isopropanol, and 200 µL of 5% Nafion solution are necessary. The solution was subjected to a sonication process for 20 min to obtain a homogeneous mixture.

### 2.2. Methodology

**Figure 1:**
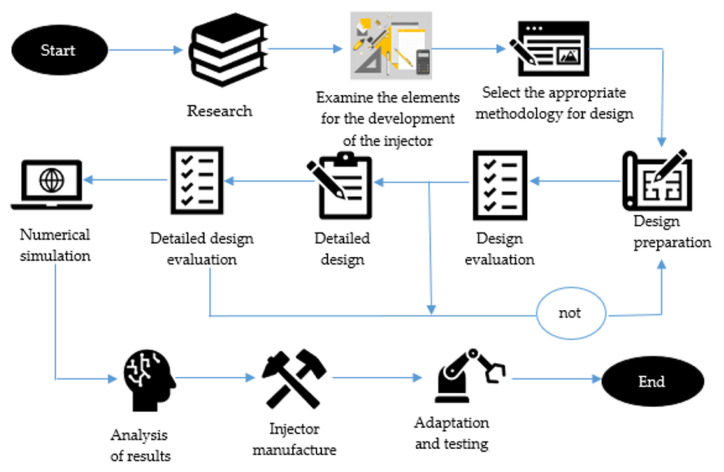
Methodology for the development of the injector.

This methodology fully describes the injector design process, consisting of information collection, management, and organization. In the development of the problem in a practical way for the design of the injector, the feasibility study, preliminary design, and detailed design were carried out. Figure 1 shows the methodology for the development of the injector.

### 2.3. Detailed Design

In developing the injector design, it is necessary to identify the needs it must cover. Therefore, the first step consists of the preparation of the design. In this step, some injector designs were made and analyzed in detail to determine whether they adapted to the needs, considering the parameters such as the dimension of the injection system, measurements where the injector will be adjusted, sizes of the printing area, injector components, and printer structure.

In the total design of the subsystems, the variables manipulated for the proper functioning of the printer and the injector must be recognized: energy, matter, and information. The energy is used to feed the mechanism used for the ink injection, the material is carbon nanotube ink, and the information is the structure to be printed.

For the design of the components, it is necessary to consider how the printer structure injector adaptation will be carried out. The components were selected according to the requirements, as in the case of the solenoid, which had a 12-V power supply and a force of 15 N. The bearing, retain, and piston adapt to the pipe diameter where they will be placed. On the other hand, the design assembly was prepared, which consisted of integrating the elements for the design of the final injector. This only consists of one piece, but it is necessary to adapt the components used for its proper functioning and its appropriate adaptation to the printer carriage.

**Figure 2:**
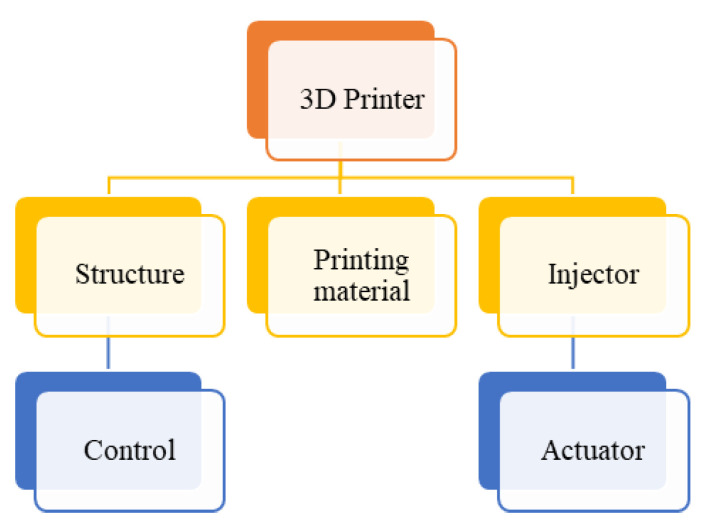
Flow chart of the 3D printer components

Once the preliminary design was achieved, considering all the parameters, prototypes were made to examine them and obtain technical approval. The prototypes were made using 3D printing, which allowed modifications to the detailed design. Finally, the injector was manufactured in aluminum alloy, which is the proper material. Figure 2 shows a chart with the main features to assemble the injector.

For the adaptation of the injector, a Cartesian coordinate printer was used. This device allows the displacement of the injector along the X, Y, and Z axes. The Ender 3^®^ printer developed by Creality, Shenzhen (China), was chosen since it works through open-source software, allowing the creation of its code instructions and controller’s firmware according to the printing needs.

For the displacement of the piston, the mechanism used was a solenoid. The solenoid JF-S0837DL was used at 12 V, with a force of 15 N that allows a displacement of 10 mm, its objective being to control the ink flow in the whole position, open or closed.

**Figure 3:**
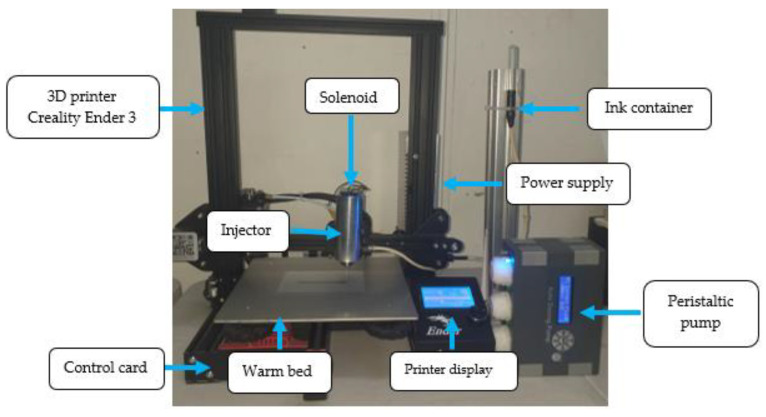
3D nano-ink printer components.

A peristaltic pump was used for the controlled dosing of the ink, being a positive displacement pump, because one part sucked and the other expelled. Its operation consisted of alternating the compression and relaxation of the hose. Internally, it had rollers that compressed the hose when it rotated and thus generated a vacuum to suck the fluid through the hose. Figure 3 shows the printer integrating the injector and the control systems used for operation.

### 2.4. Solenoid Control

The solenoid control was carried out using an Arduino Uno^®^ board, by Arduino^®^, Ivrea (Italy), which consists of a board based on a microcontroller that allows recording instructions written in a programming language. It should be noted that the programs created can interact with Arduino^®^ compatible boards.

**Figure 4:**
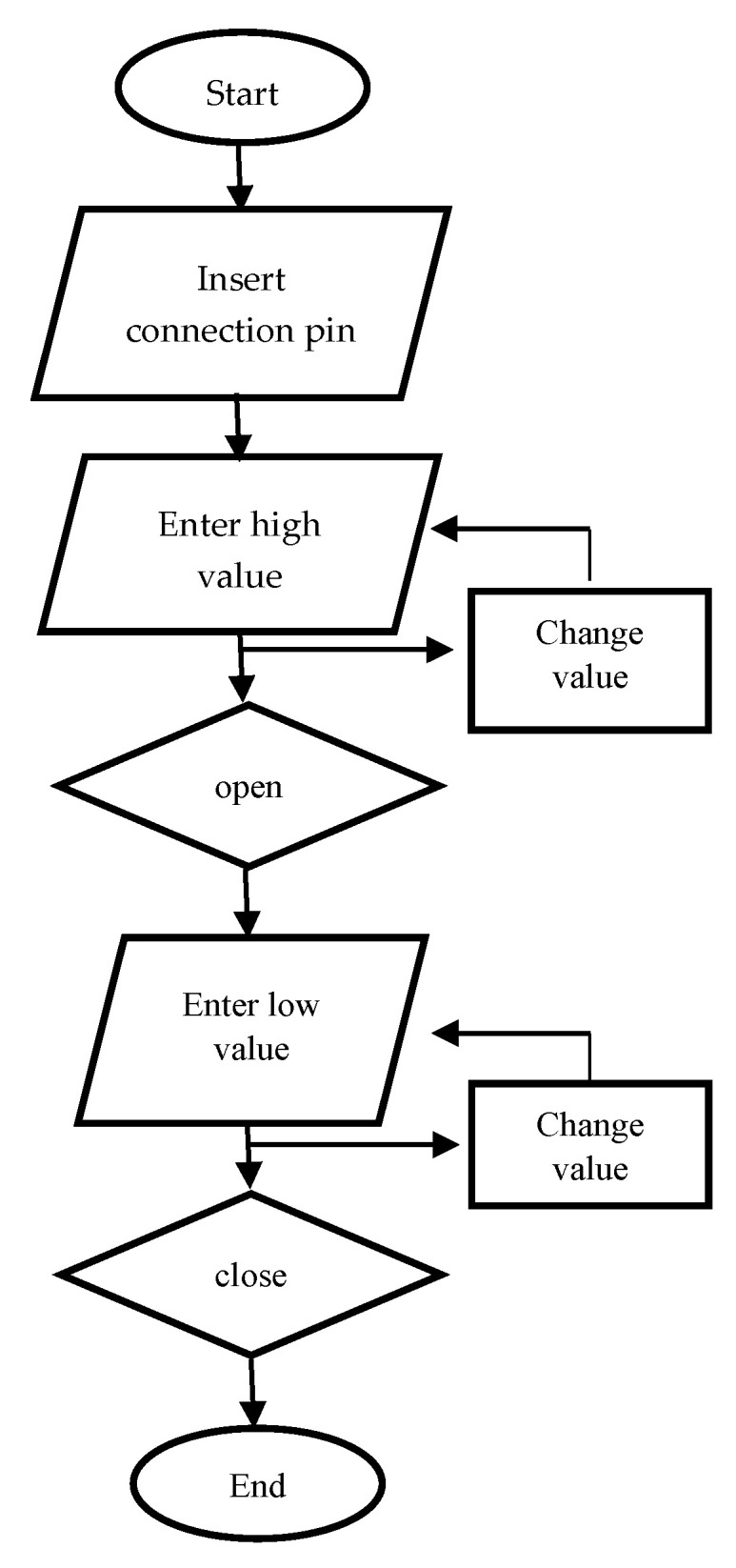
Solenoid operation flow chart.

The solenoid operation was programmed using the software Arduino IDE 2.2.1 ^®^, developed by Arduino^®^, Ivrea (Italy) and which is based on C programming. Figure 4 shows a flowchart of the process of the solenoid. The program was compiled and loaded via USB. It allows compiling and uploading the complete program to the connected card directly from the displayed window. The solenoid program is based on recording the pin used on the board when the solenoid remains open and closed, given in milliseconds.

**Figure 5:**
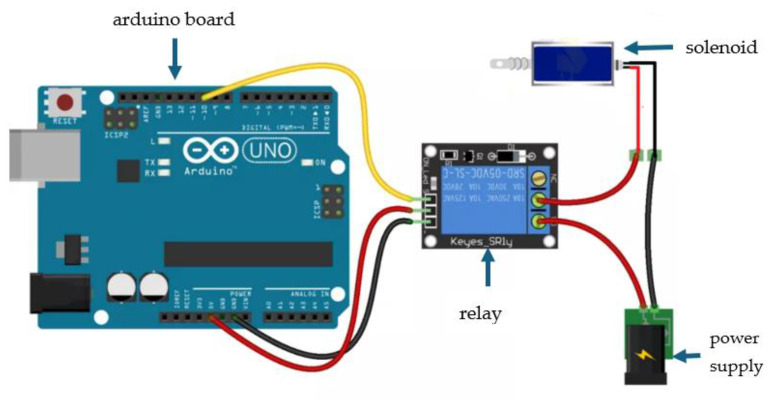
Solenoid control connection diagram.

When the program has been loaded onto the Arduino^®^ board, the operator must carry out the connection, whose diagram is shown in Figure 5, which consists of the connection of a relay that is an electromagnetic device. It is used to have control of energizing and de-energizing the coil. The solenoid is programmed on the Arduino^®^ board, depending on how fast or slow the solenoid works. It should be noted that the Arduino^®^ is powered at 5 V, while the solenoid requires a 12 V power supply; so, each device has its power supply.

### 2.5. Injector Design

**Figure 6:**
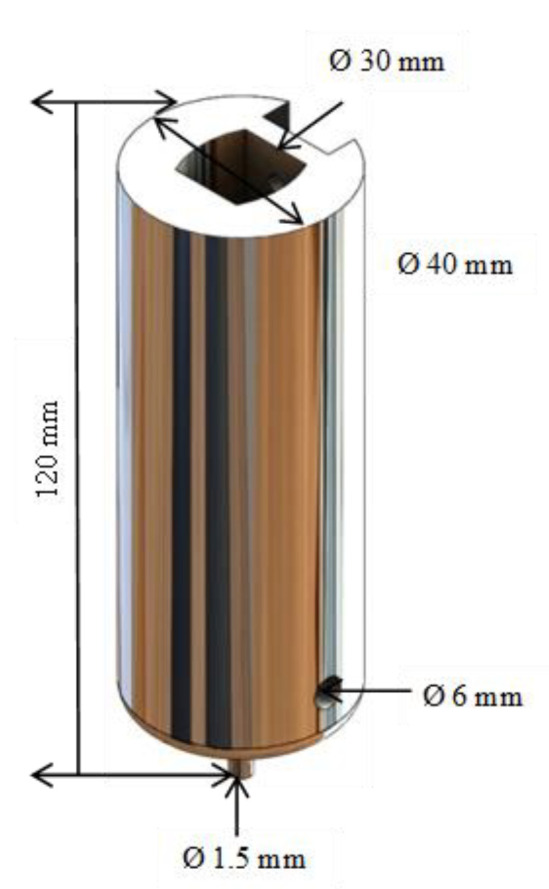
Injector design.

The injector was designed employing a computer program. The software SolidWorks^®^ 2022 developed by Dassault Systèmes, Velizy-Villacoublay (France), was chosen because it offers a simple environment for design and allows modifications without affecting the original design. When the design was adequate, considering all the analyzed parameters, it was manufactured in 7075T6 aluminum alloy and subsequently adapted to the Creality Ender 3^®^ printer. Through the prototypes modeled in 3D, many essays were developed, and we noticed that Acrylonitrile Butadiene Styrene (ABS) was not the proper material due to the carbon particles in the inner wall becoming stuck when the ink printing process was carried out. The injector manufactured in aluminum alloy avoided the stuck particles. Also, it is a low-cost alloy that allows us to manufacture it. The design parameters for the injector were the internal pipe dimensions of the injector, one of them used for the displacement of the piston and the second for the flow of the ink used as printing material. It was essential to consider the size and shape of the tip with which the ink injection was made and its adaptation. Considering the parameters, the design of the injector was made up of a single piece, and its shape was cylindrical, having a length of 120 mm and a diameter of 40 mm. Its components were located internally. Figure 6 shows the design of the injector.

**Figure 7:**
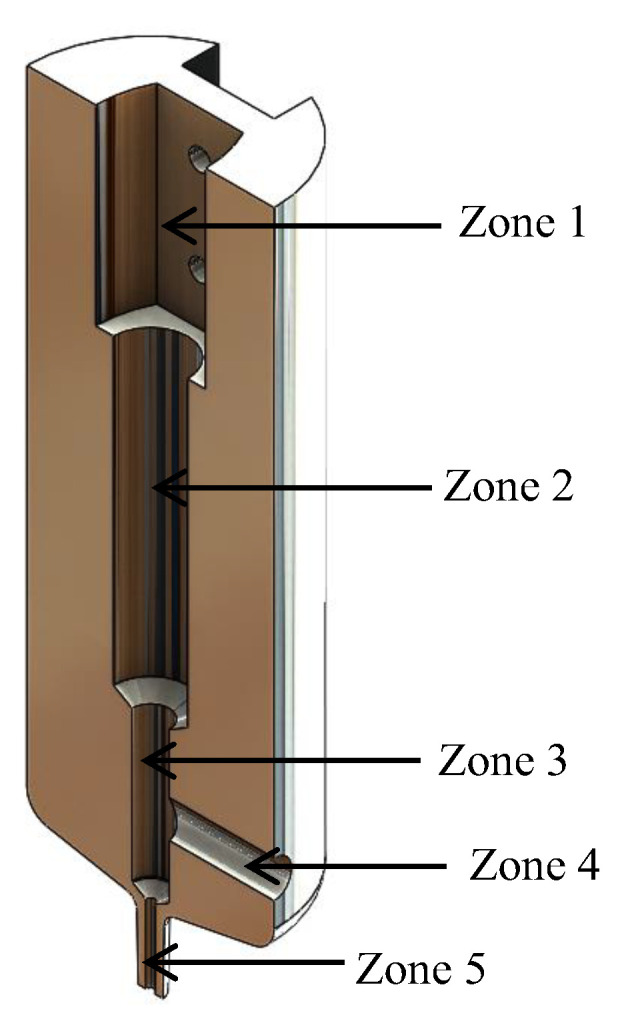
Symmetrical cut of the injector.

Internally, the injector had two pipes. The first was along the injector. Its primary function was to allow the coupling of the components internally because the measurements of the pieces were not the same. It had different diameters for its adaptation. Figure 7 shows a symmetrical section of the injector, identifying by areas how it is located internally.

Therefore, zone 1 had a diameter of 30 mm. In this zone, the adaptation of the solenoid was carried out. In zone 2, a diameter of 12 mm was used to place the linear bearing and the retainer. Zone 3 allowed the displacement of the plunger and had a diameter of 6 mm. Zone 4 was the second pipeline that made up the injector. It had a diameter of 6 mm, a connection to adapt a hose, through which the ink flowed to finally be deposited in zone 5, having a diameter of 1.5 mm, where a syringe was adapted.

**Figure 8:**
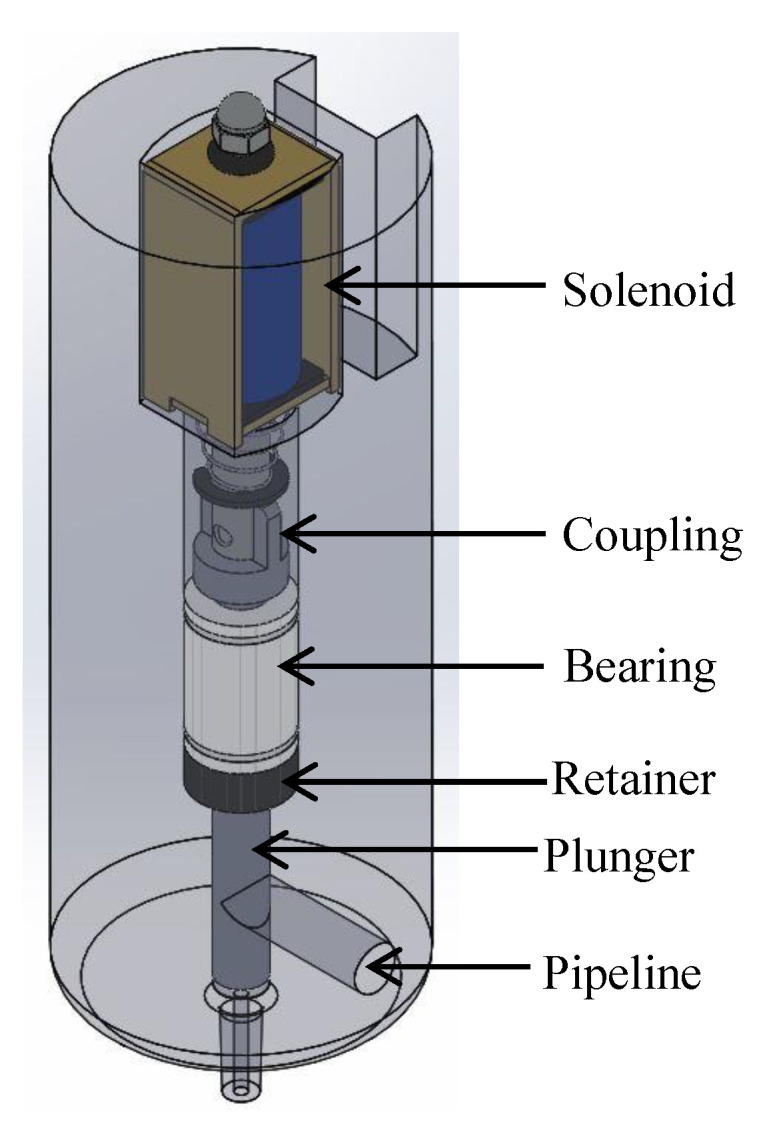
Injector with components.

Figure 8 shows the injector with the components found internally: Inside the first pipeline, the solenoid, for its adaptation to the plunger, had a coupling using a small screw and a nut. After the solenoid, there was a linear ball bearing with an internal diameter of 6 mm and a retainer. These were fixed so that they could not detach from the injector. The plunger passed through them linearly. Inside the injector was the second pipeline designed for the carbon nanotube ink to flow through so it can be deposited. Finally, a 27 G and 13 mm needle, used for ink injection, was adapted to the tip of the injector. 

The injector had a lateral connection and adapted a plastic hose to be connected to the peristaltic pump. This pump was used to control the ink. Its function was to control the fluid so that it was constant and thus allowed ink injection.

**Figure 9:**
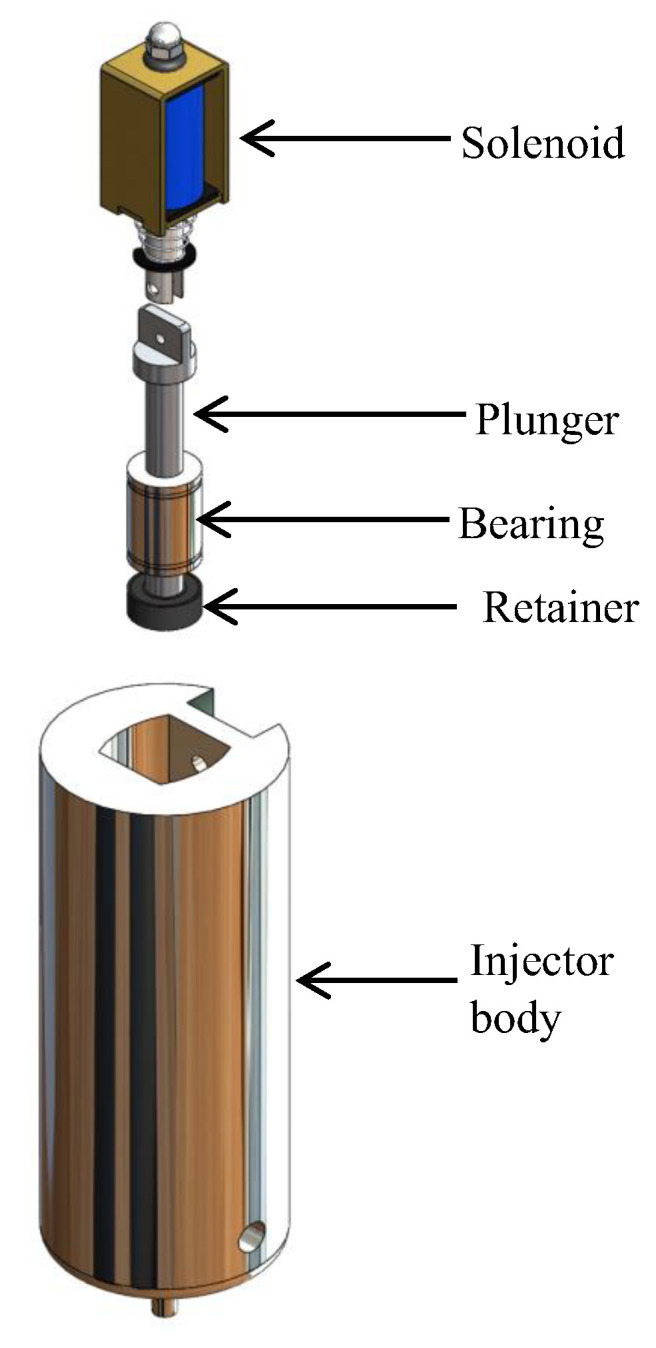
Order of assembly of the injector components.

The upper part of the injector coupled the components used. The first to be introduced was the retainer. Its function is not to allow the ink to pass its limit. After the retainer, was the linear bearing, which enables the plunger to have a linear displacement as indicated by its name. Then, internally between the bearing and the retainer, the piston moved, which had a diameter of 5.5 mm, and through a coupling was connected to the solenoid. The solenoid was electrically controlled to allow the plunger to rise and return with the aid of a spring for the passage of ink. Figure 9 shows the order of assembly of the components.

This device was designed for the ink injection of carbon nanotubes to take advantage of the properties offered by nanotubes. This device was characterized by being made up of a single piece, and its components were located internally.

## 3. Results

### 3.1. Computational Fluid Dynamics Analysis

**Figure 10:**
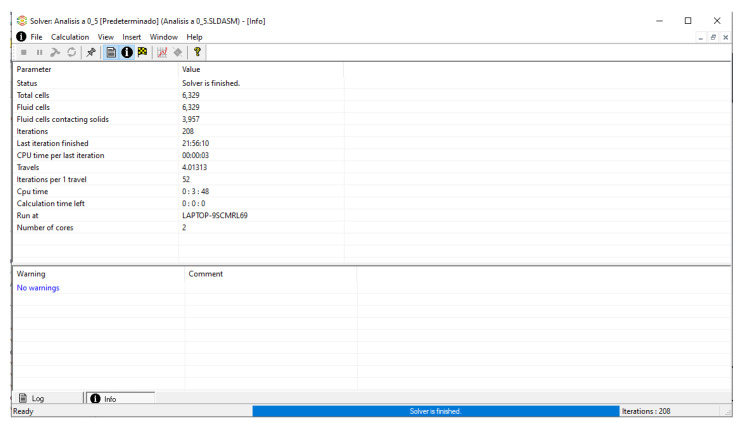
CFD solver information.

To analyze the behavior of the fluid through the injector pipes, using the SolidWorks^®^ 2022, by Dassault Systèmes, Velizy-Villacoublay (France), a Computational Fluid Dynamics (CFD) analysis was performed, which is used to predict the behavior of the fluid and to determine its characteristics when it is moving; the method to solve the fluid dynamics problem is the Finite Volume Method (FVM), and it is considered as an implicit solver for incompressible fluids and low compressibility fluids, which through iterations achieve the numerical solution. Computational fluid dynamics (CFD) analysis was performed internally to obtain values such as the pressure, velocity, and vorticity generated when the carbon nanotube ink was injected to verify whether the design met the necessary conditions. In addition, four case studies were carried out, with different distances between the plunger and the pipe through which the fluid passed. To perform computational fluid dynamics (CFD) analysis, it is essential to mention the following parameters: the type of flow used was laminar and turbulent, since both are presented in this analysis. The normal pressure and temperature conditions in which the study was carried out are added, which were 101,325 Pa and 20.05 °C. The next thing was to set the input and output boundary conditions. These are the necessary restrictions to solve a boundary value problem, a system of differential equations that solve in a domain known as a set of requirements. Then, the fluid inlet face of the model was selected, the flow rate delivered by the peristaltic pump was known, and its value was 70 mL/min; however, in m^3^/s, its value was 1.1667 × 10^−6^ m^3^/s. This value was assigned in the flow, indicating the input volumetric flow. Due to the injector geometry and to carry out the computational analysis, an automatic mesh was applied through the computational software. Once the study was solved it displayed information such as the number of iterations, cell number, and time to solve, among other data shown in Figure 10.

With the previous parameters, the analysis was finally executed, where the processor developed the numerical solution in the computer program, making several iterations until the study was entirely resolved.

#### 3.1.1. Pressure

An unknown parameter in this fluid analysis was pressure, a physical quantity defined as a force exerted over a unit area. In these case studies, the behavior of the fluid through the injector was analyzed, observing the pressure changes that occurred when the fluid passed through the injector pipes. An external force was applied, in this case, the external force was caused by the peristaltic pump when it supplied the fluid to the injector. As a result, a pressure was created that compressed the fluid. The force that was distributed by the injector pipes was the one that contributed the value to the pressure that was generated.

In this way, the volume occupied by the fluid decreases when the pressure is increased. Table 1 shows the pressure values obtained in the four studies carried out.

#### 3.1.2. Velocity

Velocity appears when the speed or slowness with which a fluid or a body moves over time is appreciated. It varies according to its magnitude and direction along its flow line, explaining it in a model that allows us to perform a detailed analysis. Below, Table 2 shows the results obtained from the research into the behavior of the fluid in its velocity parameter.

#### 3.1.3. Vorticity

Vorticity is used in fluid mechanics and is a property that quantifies the rotation experienced by a fluid. When passing to the area with a larger diameter, vorticity decreases to increase again at the injector outlet. Table 3 shows the values obtained from the vorticity generated in the case studies carried out.

These results support the geometric design; the numerical outcomes show how the internal ducts of the device allow the flow to be controlled.

### 3.2. Experimental Results

When the injector was attached to the printer, it was possible to perform tests of its operation with the carbon nanotube ink, thus obtaining the appropriate printing speed. The ink was deposited on a thermal mica to be removed from the printer’s heated bed later. To obtain a print with carbon nanotube ink, the injector and the printer must have the appropriate parameters for their operation. The reason for performing the tests was to obtain the optimal result, Table 4 shows the configuration of the experimental test.

**Figure 11:**
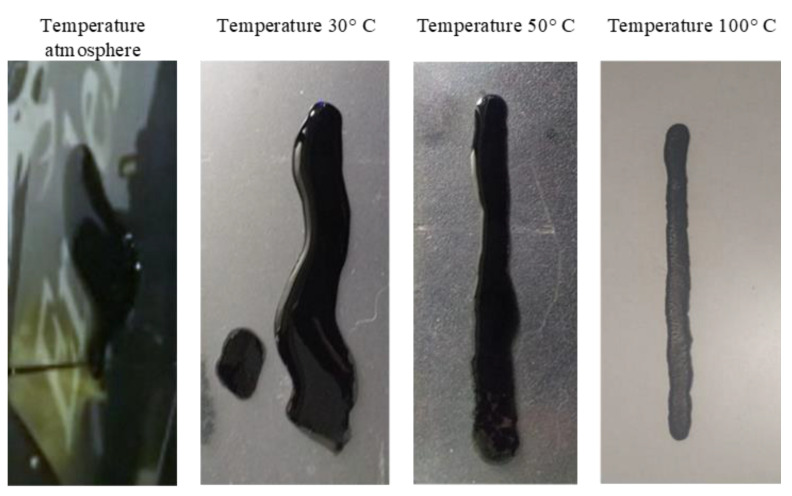
Ink printing results.

It was observed that the nanoparticles accumulated, and this sometimes caused the ink to not flow properly through the peristaltic pump, thus causing the hose used to supply the ink to become clogged. Therefore, in each test carried out, the pump had to be cleaned to avoid the accumulation of nanoparticles. The most appropriate velocity for printing was 250 ms since it allowed the ink to be injected in a controlled manner. With the help of the experimental matrix, it was possible to find the most suitable injection velocity. The next step was to find an appropriate temperature that allowed for the correct drying of the ink. For this, the heated bed made impressions with four different temperatures. In Figure 11, it can be seen that a high temperature was needed to dry the ink. In this case, the appropriate one was 100 °C, although it was not enough because there was an expansion of the ink, which did not allow the printed figure to be appropriately formed. This also indicated that the ink needed to have more viscosity because it slipped easily, complicating the figure’s printing. Finally, an amorphous figure was made at room temperature since only a jet was generated and expanded through all the mica used in print.

The temperature of 30 °C was is not enough, but it prevented the ink from expanding a lot, although it was not possible to observe the defined figure either. With a temperature of 50 °C, there was a bit more control over the ink, since the figure looked slightly more limited. At room temperature, the ink drying took a long time and more if it accumulated.

The inking process was carried out by testing the solenoid to verify the speed that suited the process and the behavior of the flow patterns at 250 ms. This speed was the optimal one; experimental tests were conducted with the temperature of the warm bed to find the proper temperature for the drying process of the ink.

**Figure 12:**
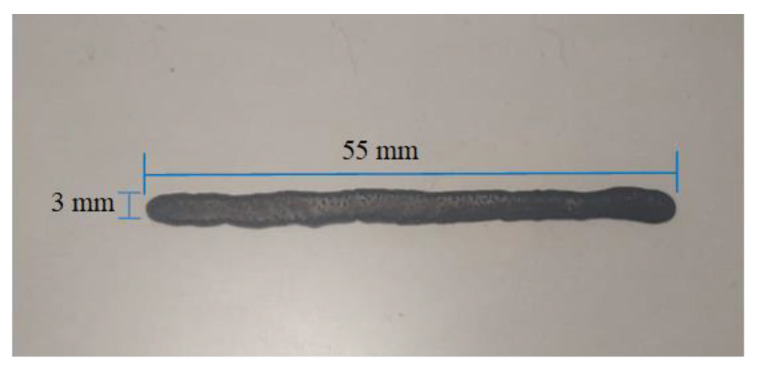
Printing sample.

Of the four prints made, the last two were the best, as they did not undergo much ink expansion and were better defined. Figure 12 shows the print made at 100 °C on the heated bed.

Analyzing the print, a line can be seen that is not perfectly defined, due to the expansion suffered by the ink, having a length of 55 mm and a width of 3 mm. The printed figure was made with an injector speed of 250 ms and a heated bed temperature of 100 °C, having an adequate result with the carbon nanotube ink according to the tests carried out.

## 4. Conclusions

The methodology was used to develop and efficiently design the injector, allowing an optimal result to be obtained through it. The final design of the injector was machined from 7075T6 aluminum alloy and adapted to the 3D printer. Through CFD analysis, the velocity, pressure, and vorticity parameters were obtained, and it was also used to anticipate the behavior of the fluid in this injection process, maintaining the indicated conditions. It obtained better results when the fluid passed through the injector without obstruction. In addition, it is a low-cost tool.

When carrying out the experimental tests of this research work, it was identified that the speed of 250 ms allowed the correct operation of the solenoid for the ink injection process, and the adequate temperature for drying the ink must be more than 100 °C.

## Figures and Tables

**Table 1:** Results of the pressure in the fluid.

Plunger Distance	
0.5 mm	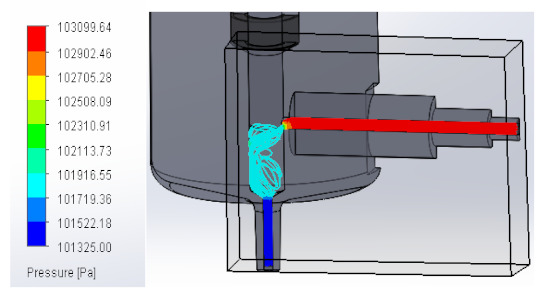
1 mm	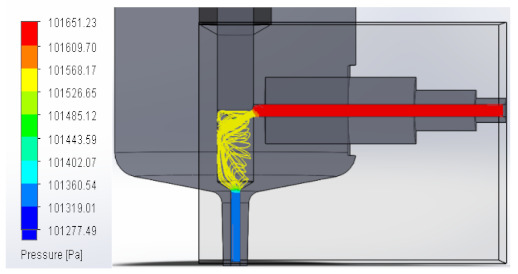
1.5 mm	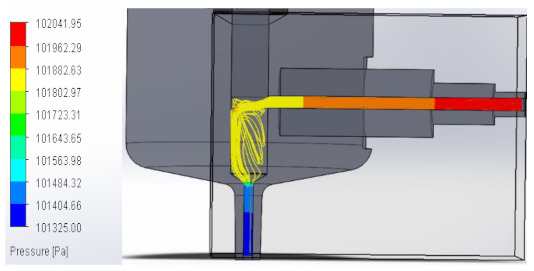
2 mm	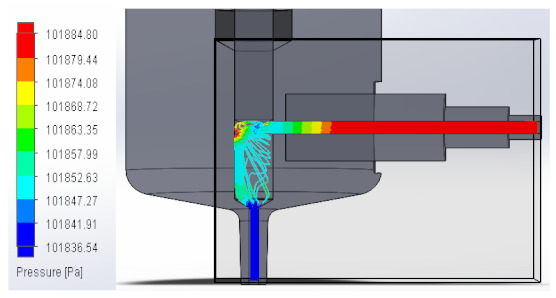

**Table 2:** Results of the velocity in the fluid.

Plunger Distance	
0.5 mm	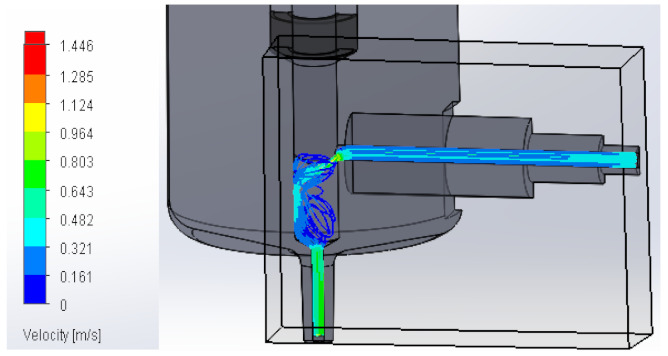
1 mm	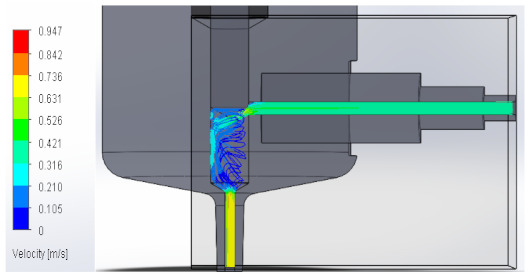
1.5 mm	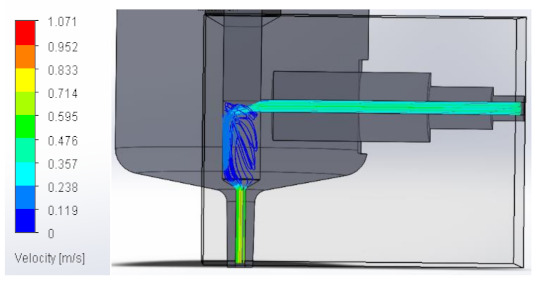
2 mm	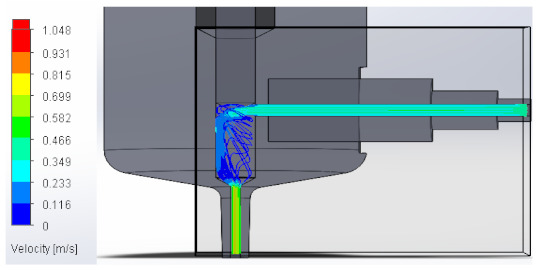

**Table 3:** Results of the vorticity in the fluid.

Plunger Distance	
0.5 mm	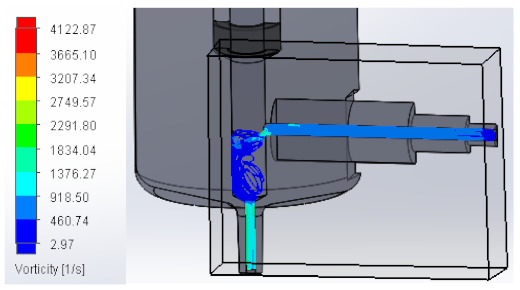
1 mm	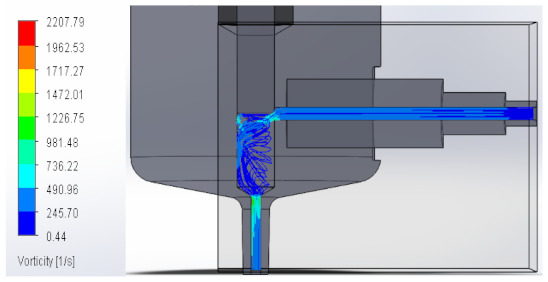
1.5 mm	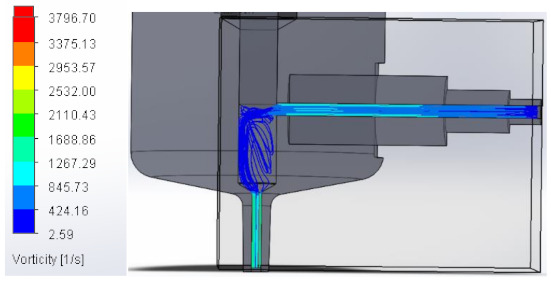
2 mm	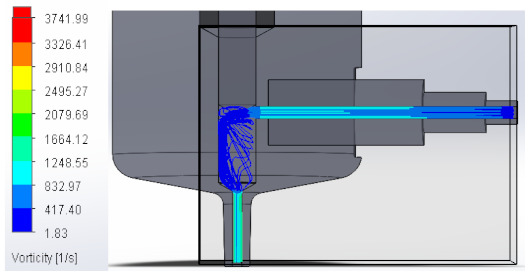

**Table 4:** Experimental matrix.

Test	Plunger Distance	Velocity (ms)	Comment
1	2 mm	50	The solenoid with this speed worked very fast. Because of this, carbon nanotube ink had a lot of turbulence and accumulation.
2	2 mm	100	The turbulence decreased, there was not much expansion of the ink, and the flow was continuous.
3	2 mm	150	Because the velocity decreased, there was no longer turbulence at the ink outlet. Therefore, there was no spreading when it is ejected.
4	2 mm	200	The ink control improved, the solenoid had slower movement, and the ink was constant without turbulence.
5	2 mm	250	The movement of the solenoid with this velocity presented better control of the ink, a constant thread was observed, and there was no turbulence at the outlet or spreading of the ink.

## Data Availability

Not applicable.
